# Comparative Surface Morphology, Chemical Composition, and Cytocompatibility of Bio-C Repair, Biodentine, and ProRoot MTA on hDPCs

**DOI:** 10.3390/ma13092189

**Published:** 2020-05-10

**Authors:** James Ghilotti, José Luis Sanz, Sergio López-García, Julia Guerrero-Gironés, María P. Pecci-Lloret, Adrián Lozano, Carmen Llena, Francisco Javier Rodríguez-Lozano, Leopoldo Forner, Gianrico Spagnuolo

**Affiliations:** 1 Department of Stomatology, Faculty of Medicine and Dentistry, Universitat de València, 46010 Valencia, Spain; james_3e@hotmail.it (J.G.); sanzjo@alumni.uv.es (J.L.S.); adrianlozano@mac.com (A.L.); llena@uv.es (C.L.); forner@uv.es (L.F.); 2 Research Group Cellular Therapy and Hematopoietic Transplant, Biomedical Research Institute, Virgen de la Arrixaca Clinical University Hospital, IMIB-Arrixaca, University of Murcia, 30120 Murcia, Spain; slg4850@gmail.com; 3 Department of Dermatology, Stomatology, Radiology and Physical Medicine, Morales Meseguer Hospital, Faculty of Medicine, University of Murcia, 30100 Murcia, Spain; juliaguerrero1@hotmail.com (J.G.-G.); mpilar.pecci@gmail.com (M.P.P.-L.); 4 Department of Neurosciences, Reproductive and Odontostomatological Sciences, University of Naples “Federico II”, 80138 Napoli, Italy; gspagnuo@unina.it; 5 Institute of Dentistry, I. M. Sechenov First Moscow State Medical University, Moscow 119146, Russia

**Keywords:** vital pulp materials, cytocompatibility, calcium silicate materials, dental pulp cells, endodontic

## Abstract

Biocompatibility is an essential property for any vital pulp material that may interact with the dental pulp tissues. Accordingly, this study aimed to compare the chemical composition and ultrastructural morphology of Biodentine (Septodont, Saint Maur-des-Fosses, France), ProRoot MTA (Dentsply Tulsa Dental Specialties, Johnson City, TN, USA), and Bio-C Repair (Angelus, Londrina, PR, Brazil), as well as their biological effects on human dental pulp cells. Chemical element characterization of the materials was undertaken using scanning electron microscopy and energy dispersive X-ray analysis (SEM-EDX). The cytotoxicity was assessed by analyzing the cell viability (MTT assay), cell morphology (immunofluorescence assay), and cell attachment (flow cytometry assay). The results were statistically analyzed using ANOVA and Tukey’s test (*p* < 0.05). EDX revealed that ProRoot MTA and Biodentine were mostly composed of calcium, carbon, and oxygen (among others), whereas Bio-C Repair evidenced a low concentration of calcium and the highest concentration of zirconium. SEM showed adequate attachment of human dental pulp cells (hDPCS) to vital pulp materials and cytoskeletal alterations were not observed in the presence of material eluates. Remarkably, the undiluted Biodentine group showed higher viability than the control group cells (without eluates) at 24 h, 48 h, and 72 h (*p* < 0.001). Based on the evidence derived from an in vitro cellular study, it was concluded that Bio-C Repair showed excellent cytocompatibility that was similar to Biodentine and ProRoot MTA.

## 1. Introduction

The dental pulp is a connective tissue formed by cells residing within a collagen-rich extracellular matrix surrounded by dentin, a mostly inorganic tubular tissue [1]. Due to their embryological, histological, and functional similarities, they are considered together as a dentin–pulp complex [2]. Upon damage, stem cells from the dental pulp (DPSCs) are recruited and induced to differentiate into odontoblast-like cells that are involved in the process of reparative tissue formation [3]. Within the field of endodontics, the different procedures involved in the preservation of pulp vitality and the promotion of its reparative potential are encompassed in the term vital pulp treatment (VPT) [4].

These procedures require the use of materials with specific biological properties, such as cytocompatibility, to ensure the survival and proliferation of stem cells present in the viable tissue with reparative potential [5]. Specifically, their chemical surface is essential in terms of cytocompatibility, as this layer will be in direct contact with living tissue and different host responses may occur in the cell–biomaterial interface [6,7]. Additionally, biomaterials used in VPT should express bioactive properties, thereby liberating calcium and hydroxide ions to form hydroxyapatite on their surface and allow for a mineral attachment to the inorganic component of dentin [8,9,10].

Mineral trioxide aggregate (MTA) was the first bioactive material used in endodontics [11], which demonstrated excellent clinical success due to its ability to stimulate pulp tissue repair and promote reparative dentin formation in early pulp wound healing [12]. However, its long setting time and discoloration potential led to the development of new biomaterials [13,14]. Silicate-based hydraulic cements were introduced in the market to alleviate the disadvantages of MTA, enhancing both its physicochemical and biological properties [15,16]. Among them, Biodentine (BD; Septodont, Saint Maur-des-Fosses, France), a tricalcium silicate cement, showed better clinical performance, less discoloration potential, and a shorter setting time than MTA [17,18,19]. Additionally, previous reports evaluating the biological effects of BD and MTA showed that BD presented accelerated dental pulp cell proliferation compared to MTA [20,21].

Bio-C Repair (Angelus, Londrina, PR, Brazil) (BCR) is a new silicate-based hydraulic cement that is presented in a ready-for-use format [22]. According to the manufacturer, it exhibits excellent consistency that is easy to be applied, acts as a barrier against microorganisms, stimulates tissue healing, and does not contribute to discoloration.

Various studies have assessed the composition, ultrastructural morphology, and biological effects of MTA and Biodentine [23,24,25]. However, to the best of the authors’ knowledge, these properties have yet to be identified and compared for Bio-C Repair. Accordingly, this study aimed to compare the chemical composition and ultrastructural morphology of ProRoot MTA, Biodentine, and Bio-C Repair, as well as the biological effects of these materials on human dental pulp cells. The null hypothesis was that there was no difference between the tested materials in their chemical composition, ultrastructural morphology, and cytotoxicity on human dental pulp cells.

## 2. Materials and Methods

### 2.1. Material Extracts

Biodentine (Septodont, Saint Maur-des-Fosses, France), ProRoot MTA (Dentsply Tulsa Dental Specialties, Johnson City, TN, USA), and Bio-C Repair (Angelus, Londrina, PR, Brazil) were prepared according to the manufacturer’s recommendations under aseptic conditions.

To characterize their composition and assess their ultrastructural morphology, three samples were prepared for each of the materials using a previously fabricated 54 × 36 × 14 mm silicone mold with a 2 mm depth and 5 mm diameter wells (Elite HD + Putty Soft Normal; Zhermack S.p.A., Badia Polesine, RO, Italy). Samples were then allowed to set for a week in an incubator at 37 °C and 95% humidity to simulate the conditions of the oral cavity.

To assess their biological effects, the materials were plated at the bottom of 12-well tissue culture plates and allowed to set for 48 h in the incubator at 37 °C and 95% humidity. Subsequently, all materials were covered with 2.5 mL of Dulbecco modified Eagle medium (DMEM) for cell culturing (Sigma-Aldrich, St. Louis, MO, USA) and incubated in the dark for 24 h at 37 °C [19]. The eluates (1:1) were prepared following the recommendations of ISO 10993-5 [26]. After incubation, 1:1, 1:2, and 1:4 eluates were filtered and added to each culture.

### 2.2. Cell Isolation and Culture

Human dental pulp cells (hDPCs) were isolated from human healthy third molars (n = 10) that were extracted for surgical reasons; this process was previously approved by the Ethical Committee of the University of Murcia (ID: 2199/2018). The pulp tissues obtained were carefully examined and immersed in 3 mg/mL collagenase type I solution (Gibco, Thermo Fischer Scientific, Waltham, MA, USA) at 37 °C in 5% CO_2_ for 90 min, as previously reported [20]. Then, single-cell suspensions were obtained and cultured in DMEM (Gibco, Thermo Fischer Scientific, Waltham, MA, USA) supplemented with 10% fetal bovine serum (FBS) (Gibco, Thermo Fischer Scientific, Waltham, MA, USA), 1% L-glutamine (Lonza, Basel, Switzerland), and 100 μg/mL penicillin/streptomycin (Sigma-Aldrich, St. Louis, MO, USA), and then incubated at 37 °C in 5% CO_2_. Cells at passages 2–4 were used for subsequent experiments.

### 2.3. Scanning Electron Microscopy (SEM): Sample Visualization, Element Analysis, and Cell Attachment

Material samples were observed under 500×, 2500×, 5000×, 10,000×, and 50,000× magnifications at 5 and 10 kV using a field emission scanning electron microscope (Hitachi S-4800, Hitachi High-Technologies Corporation, Chiyoda, Tokio, Japan). Images of the visible structural morphology were taken for posterior assessment and comparison.

The same equipment was attached to an energy dispersive X-ray analysis system (EDX; Oxford INCA 350 EDX, Abingdon, UK). The analysis was carried out at 2500×, both for areas with a regular structure and areas with a protruding structure, except for Bio-C Repair samples, in which the use of a 20,000× magnification was necessary due to their minor particle size. Three analyses were performed for each area in each of the samples, which were used to produce a table and a graph illustrating the elements present in the different areas of the samples (three samples for each material).

SEM was also used to estimate the effect of the surface chemistry of the different cements on cell adhesion and growth. A total of 5 × 10^4^ hDPCs were directly seeded to each disk surface and cultured for 72 h. Then, specimens were post-fixed with 4% glutaraldehyde in PBS for 4 h and dehydrated via a graded series of ethanol (30%–90% *v*/*v*). Before observation, samples were mounted on stubs and were coated with sputtered gold/palladium. Finally, cell attachment analysis was performed using 100×, 300×, and 1500× magnifications.

### 2.4. Cell Viability Assay

The viability of hDPCs exposed to material eluates or not (control) was analyzed using the MTT reduction assay, as described elsewhere [19], at 24, 48, and 72 h after seeding. At each time interval, 10 μL of MTT reagent (Thermo Fisher Scientific, Waltham, MA, USA) at a concentration of 5 mg/mL was added to the 90 μL growth medium/well, and then incubated at 37 °C in 5% CO_2_ for 4 h. Then, the medium was removed and dimethyl sulfoxide solution (Sigma-Aldrich, Saint Louis, MO, USA) (100 μL/well) was added and incubated for 30 min at 37 °C to dissolve the formazan crystals. Afterward, the absorbance of each well was quantified at a wavelength of 570 nm using a multi-well plate reader (Synergy H1, BioTek Instruments, Winooski, VT, USA).

### 2.5. Cell Morphology Analysis

Confocal microscopy experiments were assessed to evaluate changes in the actin cytoskeleton of hDPCs, as previously described [23]. In brief, hDPCs were seeded at a density of 1.0 × 10^4^ cells/well on 24-well plates in the culture medium containing undiluted extracts of the different cements. Untreated cells served as the control group. Then, hDPCs were fixed with 4% paraformaldehyde for 10 min, washed twice with saline solution, and permeabilized with 0.25% Triton X-100 (Sigma-Aldrich, Saint Louis, MO, USA) before fluorescent staining. Phalloidin CruzFluor™ 594 Conjugate (Santa Cruz Biotechnology, Dallas, TX, USA) and 4,6-diamidino-2-phenylindole dihydrochloride (DAPI) (Sigma-Aldrich, Saint Louis, MO, USA) were used to label the F-actin cytoskeleton and nuclei of hDPCs, respectively. Images were taken using an Axio Imager M2 Zeiss microscope (Carl Zeiss, Oberkochen, Germany).

### 2.6. Statistical Analysis

All experiments were repeated at least three times. The data obtained were analyzed using Graph-Pad Prism version 8.1.0 (GraphPad Software, San Diego, CA, USA). One-way analysis of variance (ANOVA), complemented by Tukey’s test, was applied. The level of significance was set to *p* < 0.05.

## 3. Results

### 3.1. Sample Morphology

**Figure 1:**
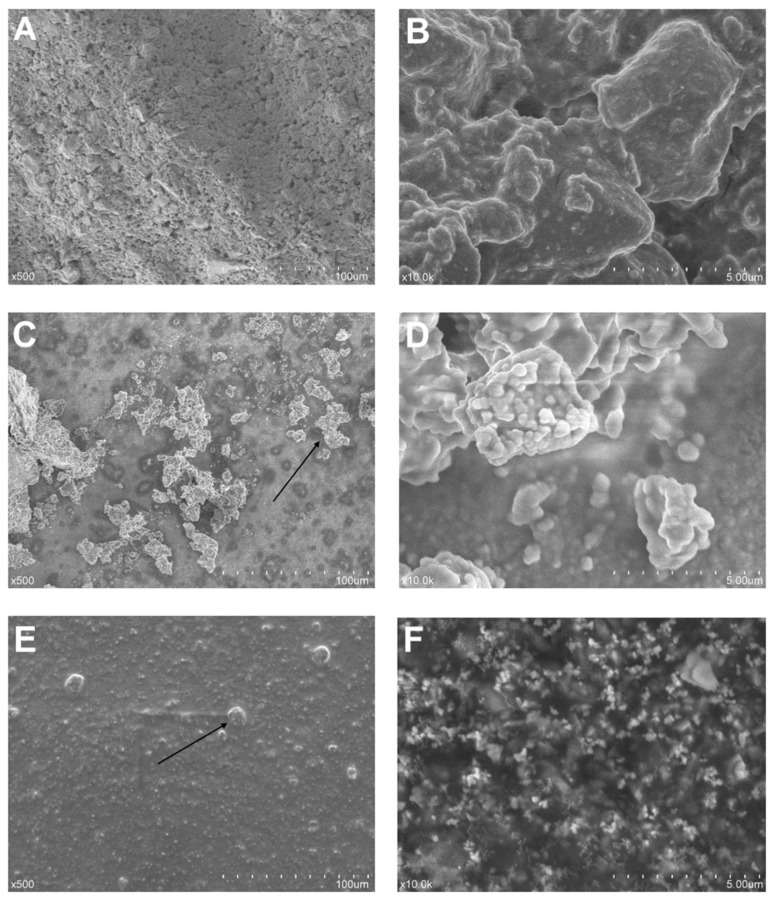
Sample morphology under scanning electron microscopy (SEM) after 1 week of setting at 37 °C and 95% humidity: (**A**) ProRoot MTA sample, 500×; (**B**) ProRoot MTA sample, 10,000×; (**C**) Biodentine sample, 500×; (**D**) Biodentine sample, 10,000×; (**E**) Bio-C Repair sample, 500×; and (**F**) Bio-C Repair sample, 10,000×. The arrows indicate exophytic areas.

SEM images of the ProRoot MTA, Biodentine, and Bio-C Repair samples under 500× and 10,000× magnification are presented in Figure 1.

ProRoot MTA exhibited a regular but non-uniform surface throughout the sample surface. Biodentine, however, showed some protruding irregular structures over a regular surface. These irregularities often appeared as clusters of small particles, while others emerged as a singular piece of material. Higher magnifications showed an ultrastructural configuration of these protruding areas similar to that of the regular areas. Bio-C Repair offered the most regular superficial morphological organization. Only a few minor protruding areas were seen. In this case, a higher magnification revealed a grainy ultrastructure.

### 3.2. Element Analysis

**Figure 2:**
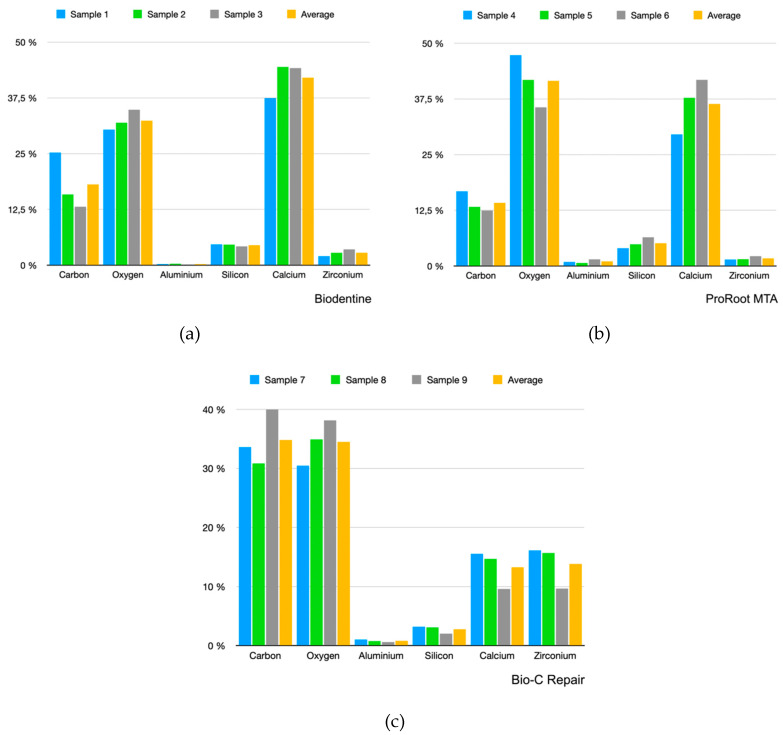
Energy dispersive X-ray (EDX) analysis results for the different samples analyzed. (**a**) Biodentine, (**b**) ProRoot MTA, (**c**) Bio-C Repair.

As explained previously, we performed three analyses per sample, obtaining nine analyses for each material. These data are represented in bar histograms (Figure 2). Table 1, Table 2 and Table 3 show the compositions of the materials obtained after the EDX analysis. For each sample, the average of the three measurements is also presented.

Carbon and oxygen showed the greatest presence within the Bio-C Repair composition. Calcium was represented in a low quantity (almost the same as zirconium, which is used as a radiopacifier), while calcium had a high abundance in the Biodentine and ProRoot MTA samples.

### 3.3. Cell Attachment Analysis

**Figure 3:**
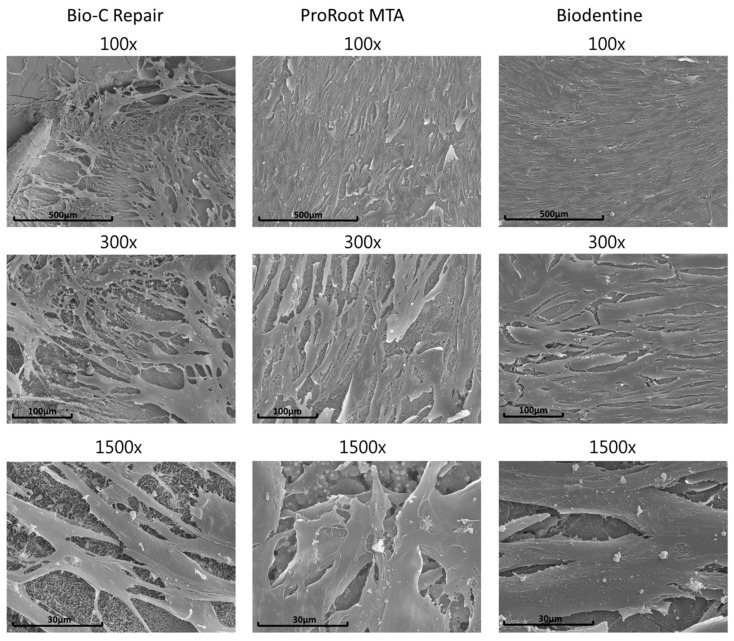
SEM images of human dental pulp cells (hDPCs) cultivated on vital pulp material (Bio-C Repair, ProRoot MTA, and Biodentine) surfaces after 72 h of culture. Magnifications of 100×, 300×, and 1500×. Scale bars: 500 μm, 100 μm, and 30 μm.

The analysis of the cell adherence and morphology of the hDPCs on the surfaces of the samples showed multilayered cultures of cells with multiple prolongations adhered to the biomaterial in all groups (Figure 3). This phenomenon confirmed the MTT assay and cell morphology results.

### 3.4. Cell Viability Assay

**Figure 4:**
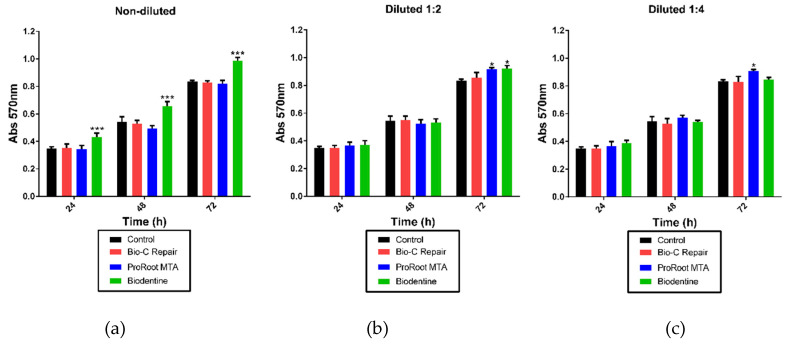
Relative formazan formation of hDPCs that were exposed to Biodentine, ProRoot MTA, and Bio-C Repair eluates, as well as the culture medium (control) for 24, 48, and 72 h. (**a**): Non–diluted, (**b**) Diluted 1:2, (**c**) Diluted 1:4. The bar heights represent the mean values and the line extensions represent the standard deviations. Significant differences compared to the control are marked with an *, where * means *p* < 0.05, and *** means *p* < 0.001.

The cytotoxic effects of the three materials over 24, 48, and 72 h are summarized in Figure 4. The relative formazan production evidenced that Biodentine, Bio-C Repair, and ProRoot MTA were not cytotoxic to hDPCs. Remarkably, the undiluted Biodentine exhibited significantly higher levels of relative formazan formation than the control group (without eluates) at 24 h, 48 h, and 72 h (*p* < 0.001). All dilutions tested in the Bio-C Repair group presented similar formazan production compared with untreated cells (control). Concerning the ProRoot MTA group, the 1:2 and 1:4 dilutions presented higher formazan formation than the control group at 72 h (*p* < 0.05).

### 3.5. Cell Morphology Analysis

**Figure 5:**
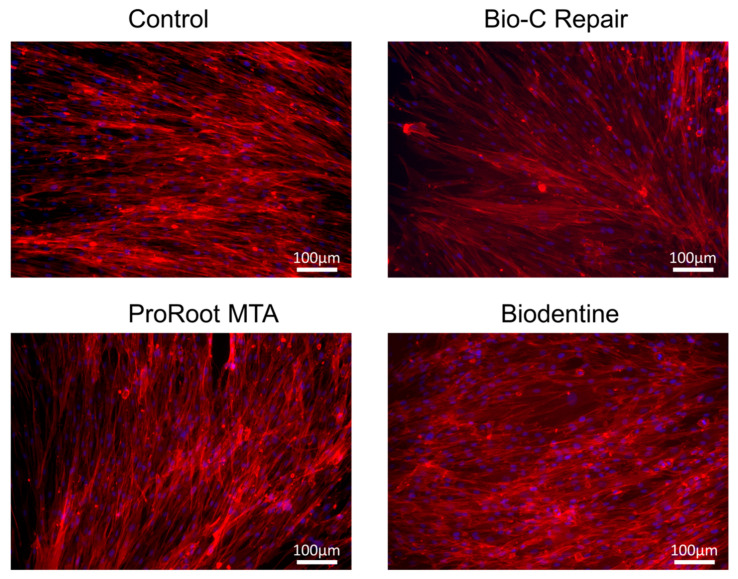
Confocal analysis was used to analyze changes in the actin cytoskeleton of hDPCs exposed to material eluates. The confocal microscopy images show F-actin labeling with CruzFluor 594-conjugated phalloidin (red) and nuclei with DAPI (blue). Scale bar: 100 μm.

After 72 h of exposure of the cell cultures to the eluates of different materials, hDPCs showed an extended morphology and similar well-organized F-actin filaments compared to the control group. The presence of aberrant cells with a pyknotic nucleus, reflecting the apoptotic or necrotic status of the cells, was not evidenced (Figure 5).

## 4. Discussion

Biocompatibility has been defined as the ability of a material or a substance to perform with an appropriate host response when applied as intended. Furthermore, it is an essential aspect that should be considered when selecting a material for direct contact with vital tissues [27,28].

Scanning electron microscopy (SEM) is a commonly used technique for the observation of the microstructure of biomaterials [29,30], along with the EDX system for element analysis [31,32]. The dimensions of the samples used in the present study were described in other studies assessing silicate-based materials [20,33], and are also similar to those used in other studies in the field [22,34,35,36]. Samples were stored at 37 °C to allow for the complete setting of the biomaterials. This temperature was maintained for a week, aiming to simulate clinical conditions, unlike other studies, which kept these conditions for only 24–48 h [20,33,34].

Results from the element analysis revealed that ProRoot MTA was mostly composed of oxygen (41.58%) and calcium (36.37%). Similarly, Biodentine showed high levels of oxygen (32.40%) and calcium (42.03%). This coincides with the results shown by Camilleri et al. [31], except for the concentration of silicon shown, which was lower in our study (4.49% vs. 9.2%). These two materials exhibit a similar composition [37]. However, Bio-C Repair was mostly composed of carbon (34.81%) and oxygen (34.51%), with a lower concentration of calcium compared to the other biomaterials. This could be associated with its clinical application as a repair material, as the same version of the biomaterial designed as an endodontic sealer (Bio-C Sealer) shows a higher concentration of calcium in its composition [38]. The higher calcium content in Bio-C Sealer may be explained by the phenomenon of bioactivity. As described by previous studies, calcium-silicate-based cements are capable of releasing their major cationic components, resulting in the precipitation of a hydroxyapatite-like mineral on their surface. The formation of a mineral attachment to the inorganic content of dentine is a desirable property among endodontic cements, which aim to seal viable tissue with reparative potential from possible harm [39,40].

Additionally, Biodentine and Bio-C Repair both showed higher levels of zirconium compared to ProRoot MTA, as they use zirconium oxide as a radiopacifier in contrast with bismuth oxide in ProRoot MTA. Our results for the general elemental composition, found for both ProRoot MTA and Biodentine, concurs with that from a microstructural analysis carried out by Ashofteh Yadzi et al. [37] in 2019, except for those elements with a low proportion within the materials’ composition, which were considered in their study as “trace elements.” However, another study [41] reported the presence of phosphorous in both ProRoot MTA and Biodentine using energy dispersive spectroscopy (EDS). Nonetheless, minor differences in the expected chemical composition reported in EDX/EDS analyses can be produced as a result of variances between the equipment used and the measurements carried out. The composition results reported in the present study were based upon the mean result from the three measurements that were carried out for each of the samples.

Furthermore, studies have also identified the chemical composition of ProRoot MTA using X-ray diffraction to detect the chemical compounds and crystalline composition. In 2006, Song et al. [42] used this methodology to detect phosphorous-containing compounds (i.e., calcium phosphate, magnesium phosphate, barium zinc phosphate) in ProRoot MTA’s powder, while no phosphorous was found once the material was set. The EDX analysis performed in the present study was also carried out after the setting period of the materials, thus coinciding with the results shown by Song et al.

When comparing the composition of Biodentine, both in the regular and protruding areas observed under SEM, no differences were observed. Therefore, we confirmed that the study samples had the same composition throughout their surface, but different ultrastructural morphologies. Interestingly, Bio-C Repair showed a greater aluminum content in protruding areas compared to regular areas.

To simulate the clinical situation of vital pulp materials placed on remaining dentine thicknesses of 0.01 to 0.25 mm or in cavities with pulp exposure, where there are fluids, the evaluation of these materials was carried out via incubation of the cultured cells with several extracts or eluates of the materials (1:1, 1:2, and 1:4) according to the International Standard ISO 10993-5 [26]. The MTT formazan method is a cytotoxicity test widely used in the literature that evaluates the possible changes in the cellular functions that might lead to damage to cellular survival and function [43]. According to the present MTT assay results, the test found no statistically significant differences between the vital pulp materials, and none of them exhibited cytotoxic effects on hDPCs. Previous reports have shown different results with MTA-based materials. Youssef et al. [25] reported that formazan production in the presence of ProRoot MTA was 53%, whereas other authors showed that ProRoot MTA eluates induced significant proliferation of DPSCs [44] and adequate cell viability of human mesenchymal stem cells from bone marrow [45]. Regarding Bio-C Repair, Benetti et al. [22] demonstrated that Bio-C Repair is a premixed and biocompatible material that induces mineralization in vivo. Finally, excellent cytocompatibility was observed with Biodentine. Our results showed that Biodentine displayed lower cytocompatibility when compared to untreated cells and the other vital pulp materials. These results are consistent with recently published studies [46,47].

Furthermore, the biological reaction of cells in contact with these materials was evaluated using direct contact (SEM) and indirect contact (immunofluorescence assay) methods. Cell morphology, when attached to the surface of a biomaterial, can be a predictable sign of cell function and differentiation and the chemical compositions of materials can influence the cytotoxicity toward hDPSCs [48]. Additionally, previous reports have shown that tricalcium silicate-based cements that are in contact with tissue fluids produce calcium hydroxide that releases calcium ions, favoring the migration of stem cells and differentiation into odontoblast-like cells, which are two essential processes involved in pulpal repair [20,23]. The results of this study showed adequate attachment of hDPCS to vital pulp materials, where multilayered cultures of cells with multiple prolongations adhered to the biomaterial were evidenced. It has been reported that cytotoxic dental materials induce shrinkage of the actin cytoskeleton, pyknotic nuclei (predominantly rounded in shape), and a reduction in adherent cells [49]. Cytoskeletal alterations were not observed in the presence of material eluates. These findings also agree with previous studies, which indicated low cytotoxicity of vital pulp materials [47,50].

## 5. Conclusions

Based on the evidence derived from an in vitro cellular study, it was concluded that Bio-C Repair exhibited excellent cytocompatibility that was similar to Biodentine and ProRoot MTA. These results suggest that Bio-C Repair can be used as a vital pulp material.

## Figures and Tables

**Table 1:** Element analysis for the Biodentine samples.

Element	Sample 1	Sample 2	Sample 3	Average
Carbon	25.25%	15.88%	13.10%	18.08%
Oxygen	30.39%	31.97%	34.84%	32.40%
Aluminium	0.23%	0.31%	0.16%	0.23%
Silicon	4.67%	4.61%	4.19%	4.49%
Calcium	37.46%	44.45%	44.19%	42.03%
Zirconium	2.02%	2.78%	3.53%	2.78%

**Table 2:** Element analysis for the ProRoot MTA samples.

Element	Sample 4	Sample 5	Sample 6	Average
Carbon	16.76%	13.29%	12.42%	14.16%
Oxygen	47.32%	41.80%	35.63%	41.58%
Aluminium	0.91%	0.69%	1.50%	1.03%
Silicon	4.01%	4.89%	6.46%	5.12%
Calcium	29.55%	37.77%	41.78%	36.37%
Zirconium	1.44%	1.55%	2.21%	1.73%

**Table 3:** Element analysis for the Bio-C Repair samples.

Element	Sample 7	Sample 8	Sample 9	Average
Carbon	33.61%	30.85%	39.98%	34.81%
Oxygen	30.48%	34.92%	38.14%	34.51%
Aluminium	1.01%	0.75%	0.60%	0.79%
Silicon	3.20%	3.08%	2.00%	2.76%
Calcium	15.56%	14.71%	9.60%	13.29%
Zirconium	16.14%	15.69%	9.67%	13.83%
